# Transcriptome Analysis Identifies Signaling Pathways Related to Performance, Meat Quality, and Organ Development in Multienzyme‐Supplemented Kampung Unggul Balitbangtan (KUB) Chickens

**DOI:** 10.1155/vmi/2822518

**Published:** 2026-04-15

**Authors:** Siti Rani Ayuti, Mirni Lamid, Sunaryo Hadi Warsito, Mohammad Anam Al Arif, Eun Joong Kim, Sangsu Shin, Iwan Sahrial Hamid, Maslichah Mafruchati, Muslim Akmal, Aswin Rafif Khairullah

**Affiliations:** ^1^ Doctoral Program of Veterinary Science, Faculty of Veterinary Medicine, Universitas Airlangga, Jl. Dr. Ir. H. Soekarno Kampus C Mulyorejo, Surabaya, 60115, East Java, Indonesia, unair.ac.id; ^2^ Laboratory of Biochemistry, Faculty of Veterinary Medicine, Universitas Syiah Kuala, Jl. Tgk. Hasan Krueng Kalee No. 4 Kopelma Darussalam, Banda Aceh, 23111, Aceh, Indonesia, unsyiah.ac.id; ^3^ Division of Animal Husbandry, Faculty of Veterinary Medicine, Universitas Airlangga, Jl. Dr. Ir. H. Soekarno Kampus C Mulyorejo, Surabaya, 60115, East Java, Indonesia, unair.ac.id; ^4^ Department of Animal Science and Biotechnology, Kyungpook National University, Sangju, 37224, Republic of Korea, knu.ac.kr; ^5^ Division of Basic Veterinary Medicine, Faculty of Veterinary Medicine, Universitas Airlangga, Jl. Dr. Ir. H. Soekarno Kampus C Mulyorejo, Surabaya, 60115, East Java, Indonesia, unair.ac.id; ^6^ Division of Veterinary Anatomy, Faculty of Veterinary Medicine, Universitas Airlangga, Jl. Dr. Ir. H. Soekarno Kampus C Mulyorejo, Surabaya, 60115, East Java, Indonesia, unair.ac.id; ^7^ Laboratory of Histology, Faculty of Veterinary Medicine, Universitas Syiah Kuala, Jl. Tgk. Hasan Krueng Kalee No. 4 Kopelma Darussalam, Banda Aceh, 23111, Aceh, Indonesia, unsyiah.ac.id; ^8^ Research Center for Veterinary Science, National Research and Innovation Agency (BRIN), Jl. Raya Bogor Km. 46 Cibinong, Bogor, 16911, West Java, Indonesia, brin.go.id

**Keywords:** feed, gene expression, KUB chicken, meat quality, performance

## Abstract

Kampung Unggul Balitbangtan (KUB) chicken is a superior Indonesian native breed with high adaptability, good growth potential, and desirable meat quality. Nutritional strategies such as multienzyme supplementation are increasingly applied to improve productivity and meat quality without altering basal diet composition. This study investigated the effects of dietary multienzyme supplementation, consisting of phytase and protease, on growth performance, carcass characteristics, organ development, meat quality, and muscle‐related gene expression in KUB chickens. Sixteen one‐day‐old male KUB chicks were randomly allocated to four dietary treatments: a control diet without multienzymes and three diets supplemented with phytase (200 mg/kg) combined with protease at 300, 500, or 700 mg/kg of feed. Growth performance parameters were measured up to 45 days of age, followed by carcass evaluation, organ weight analysis, meat quality assessment, and transcriptomic and quantitative real‐time PCR analyses of breast muscle tissue. Multienzyme supplementation significantly improved final body weight and feed conversion ratio, particularly during the finisher phase, while reducing feed intake at higher enzyme inclusion levels. Carcass percentage and breast weight were enhanced in multienzyme‐treated groups, accompanied by favorable changes in digestive and immune organ development. Meat quality analysis showed increased water‐holding capacity, reduced cooking loss, and improved color characteristics, notably higher redness and lower yellowness values. Transcriptomic profiling and gene expression analysis revealed significant modulation of key muscle growth‐related genes, including ACTA1, MYBPC1, TGFβ2, IGF2, and MYH9, indicating adaptive transcriptional responses associated with improved nutrient utilization rather than direct structural muscle alterations. In conclusion, dietary supplementation with a combination of phytase and protease effectively enhances growth performance, feed efficiency, carcass traits, and meat quality in KUB chickens while modulating growth‐related gene expression. Multienzyme inclusion represents a promising and cost‐effective nutritional strategy for improving productivity and meat quality in native chicken production systems.

## 1. Introduction

Products derived from chicken meat play a critical role in human nutrition, and genetic selection has been widely applied to enhance both the quantity and quality of poultry meat production [1]. The poultry industry continues to face the challenge of improving meat quality while maintaining optimal growth performance [2]. As the global population expands, increasing attention has been directed toward meeting the rising demand for animal‐derived protein sources [3]. In this context, native Indonesian chicken breeds contribute substantially to the animal protein supply chain and play an important role in supporting rural livelihoods and national economic development. Kampung Unggul Balitbangtan (KUB) chicken is gaining popularity due to its low feed conversion ratio (FCR), which is in line with consumer preferences and excellent performance [4]. KUB chickens have many advantages, such as large bodies, extraordinary meat quality, environmental adaptability, and high disease resistance compared to ordinary free‐range chickens, although this does not mean they are completely immune to disease, making them known as superior chickens [5, 6].

Dietary supplementation of broiler chickens with a multienzyme combination consisting of phytase and protease has been widely applied to improve growth performance by enhancing phosphorus and protein digestibility while mitigating the antinutritional effects of feed components such as phytate and trypsin inhibitors [7, 8]. Numerous studies have demonstrated that the inclusion of enzyme mixtures containing phytase and protease significantly improves growth performance and nutrient utilization in poultry [9]. Although responses to multienzyme supplementation vary across studies, with some trials reporting significant improvements and others showing positive trends, the overall evidence supports the beneficial role of multienzyme additives in poultry nutrition [10]. Multienzyme supplementation has been reported to improve carcass traits and meat quality in poultry. The selection of phytase and protease in this study is based on their complementary functions: phytase enhances phosphorus bioavailability by reducing phytate–mineral complexes, while protease improves protein digestion and amino acid availability by alleviating the effects of endogenous protease inhibitors [11]. Their combined use is therefore expected to synergistically enhance nutrient utilization efficiency. In KUB chickens, this strategy is anticipated to improve productivity in a cost‐effective manner and to facilitate the determination of an optimal multienzyme inclusion level without altering the basal nutrient composition of the diet [5, 10].

KUB chicken is one of the leading native Indonesian breeds, characterized by high adaptability and genetically improved traits resulting from selective breeding programs [12]. Marker‐assisted selection (MAS) has been successfully applied to accelerate genetic improvement by identifying candidate genes closely associated with growth‐related traits in KUB chickens [13]. Previous studies have reported that genes encoding actin alpha 1 (ACTA1), myosin‐binding protein C1 (MYBPC1), insulin‐like growth factor 2 (IGF2), transforming growth factor beta 2 (TGFβ2), and myosin heavy chain 9 (MYH9) are predominantly expressed in muscle tissue and play important roles in muscle development, organ growth, and meat quality attributes [14]. It is expected that this field will continue to use transcriptomic technologies to identify new candidate genes or miRNAs associated with specific phenotypes [15]. In this context, breast muscle tissue from KUB chickens has been used for miRNA‐sequencing (miRNA‐seq) at 60 days of age in subsequent studies [16]. This study aimed to investigate to determine the effect of multienzyme supplementation in KUB chicken feed on production performance and gene expression in relation to increased growth.

## 2. Materials and Methods

### 2.1. Research Design

Four dietary treatments differing in multienzyme supplementation levels were evaluated in this study. All experimental diets were formulated to meet or exceed established poultry nutritional requirements. The feeding program was divided into two phases, starter and grower, and was applied from hatch until 15, 30, and 45 days of age. Throughout the experimental period, chickens were housed individually in battery cages under a closed‐system management to allow precise control of feed intake (FI) and minimize variation among experimental units. The four treatments consisted of 0 without multienzyme (control or SR) and a combination of phytase enzyme 200 mg/kg and protease enzyme 300 mg/kg each (SRA), a combination of phytase enzyme 200 mg/kg and 500 mg/kg (SRB), and a combination of phytase enzyme 200 mg/kg and 700 mg/kg (SRC), according to the formulation of the feed ingredients. The multienzyme supplements used in this study were phytase enzyme and protease enzyme (Pharmaceuticals Co., Ltd, Shanghai, China). A total of 16 one‐day‐old male KUB chicks were obtained from a commercial breeder and randomly allocated to four dietary treatments (*n* = 4 birds per treatment). Each individual bird served as an independent biological replicate for growth performance and organ development analyses. Feed and water were provided ad libitum. A subset of birds from each treatment was subsequently used for transcriptomic analysis, as described in the RNA‐seq experimental design section.

### 2.2. Sample Preparation

At the end of the experiment, 16 chickens from each treatment were weighed and fasted for 10 h before slaughter. Slaughtered chickens were water scalded, feathers were mechanically plucked in a rotary drum plucker, and feathers were discarded. After removing the heads, necks, shanks, and legs, the carcasses were promptly weighed to determine the postslaughter carcass weight sans offal. The liver, heart, and gizzard were among the offal that was taken out and weighed. For additional molecular analysis, each component was weighed independently and put in sealed plastic bags [17].

### 2.3. Animal Euthanasia Procedure

Before sample collection, birds were humanely euthanized to minimize pain and distress. Each chicken was anesthetized with an intramuscular injection of ketamine hydrochloride (Ketamine HCl, 20 mg/kg body weight; Zoetis, Parsippany, NJ, USA) combined with xylazine (2 mg/kg body weight; Bayer AG, Leverkusen, Germany). After confirmation of deep anesthesia (absence of corneal reflex and response to toe pinch), euthanasia was completed by cervical dislocation performed by trained personnel. All procedures were conducted in accordance with institutional animal care and use guidelines and followed approved ethical standards.

### 2.4. RNA Extraction and Real‐Time PCR

CPT‐I, ACC, and avANT gene expression was measured using two‐step quantitative real‐time reverse transcription‐PCR (RT‐PCR). TRIzol reagent (Qiagen, Hilden, Germany) was used to extract the liver’s total RNA in accordance with the manufacturer’s instructions. In accordance with the manufacturer’s instructions, 2 μg of total RNA were reverse transcribed to cDNA using an Omni script Reverse Transcription Kit (Qiagen, Germany). All RT‐PCR reactions were conducted using an RT‐PCR detection system (Eco system, PCRmax, Staffordshire, United Kingdom) with a final volume of 20 μL of SYBR green real‐time PCR Master Mix Plus (Solis BioDyne, Tartu, Estonia) in 48‐well plates. Table 1 displays the sense and antisense primers’ oligonucleotide sequences. Three duplicates of each measurement were made, and average results were found. The standard calibration curves that were performed concurrently with the samples were used to quantify a standard curve. GAPDH mRNA served as the internal reference for all quantifications, and each primer set also included a negative control or no sample. The values were represented as the ratio of GAPDH mRNA values in arbitrary units after being normalized with GAPDH mRNA expression [18]. Furthermore, the ideal quantity and reference genes needed for trustworthy normalization of RT‐qPCR data were ascertained using genome.

**TABLE 1:** Primer sequences of the related genes assessed in this study.

Gene	Primer sequence	Annealing temperature	Reference/acc. number
ACTA1	F: 5′ ACT GGG ACG AGA TGG AGA AG 3′	58°C	NC_006090.3
	R: 5′ TCC AGA GCC ACA TAG CAC AG 3′		

MYBPC1	F:5′ CAC​GGT​GGA​TGA​GGC​TGA​AT 3′	58°C	XM_025155757.1
	R:5′ CTG​CTC​CAA​TGT​GGT​CTG​GT 3′		

TGFβ2	F: 5′ GCC ATA GGT TCA GTG CAA G 3′	58°C	X58071
	R: 5′ TGA CAG AAG CTC TCA AGC C 3′		

IGF2	F: 5′ GCT GGG GAC CCA ATA GAA CC 3′	56°C	NC_006092.3
	R: 5′ TCC CCA GGA GAT CAC AAA TCG 3′		

MYH9	F: 5′ GCA GTA GAG GCC AGA AAG AA 3′	57°C	XM_046906961.1
	R: 5′ AGG TCC ACA GCG ATG TCA TC 3′		

### 2.5. Screening and Functional Analysis of DEGs

Transcriptome profiling was performed to analyze gene expression patterns in the breast muscle of KUB chickens. Differential expression analysis was conducted using a statistical model, and genes were defined as differentially expressed when they exhibited an absolute fold change ≥ 2 and a false discovery rate (FDR)–adjusted *p* value < 0.05. Multiple testing correction was applied using the Benjamini–Hochberg method. Genes that did not meet both criteria were excluded from further analyses. Functional enrichment analyses of DEGs and positively selected genes (PSGs) were performed using the Kyoto Encyclopedia of Genes and Genomes (KEGG), and Gene Ontology (GO) databases via the KEGG Orthology‐Based Annotation System (KOBAS) 3.0, with statistical significance set at an adjusted *p* value < 0.05 [19].

### 2.6. Production and Meat Quality Parameters

Weekly measurements of the following production parameters were made: body weight (BW), body weight gain (BWG), FI, and FCR. Every day, mortality was noted and tracked and the body weight of each dead chicken was modified for FI and FCR [20]. Meat quality parameters measured included water‐holding capacity (WHC), pH, texture, and cooking loss (CL) measured using 5‐g raw meat initial weight samples (2 samples per replication). The meat shear force was measured by cutting cooked meat portions into six cores (20 × 13 × 13 mm) on each muscle sample with a diameter of 1.25 cm. Each sample’s color was measured using a colorimeter (12 mM Aperture U 59730‐30, Cole‐Parameter International Inc., Pittsford, NY, USA) on the same spot. Each sample’s color was represented by the meat’s lightness (*L* ^∗^), redness (*a* ^∗^), and yellowness (*b* ^∗^) values [21].

### 2.7. Statistical Analysis

Analysis of variance (ANOVA) was performed on the data using SAS’s General Linear Model (GLM) technique (SAS Institute Inc., Cary, NC). Orthogonal polynomials were used to evaluate the multienzyme level’s exponential, linear, and quadratic effects. The experimental unit was the duplicate. For the variable of interest, the SEMs computed using SAS’s GLM technique were averaged to determine the pooled standard error of the mean (SEM). *p* values less than 0.05 were regarded as statistically significant.

## 3. Results

### 3.1. Functional, GO, and KEGG Pathway Enrichment Analysis

Figure 1 illustrates the results of KEGG pathway enrichment analysis, indicating that several metabolic and muscle growth–related pathways are significantly associated with transcriptional variation potentially linked to meat quality. As shown in Figure 1, the enrichment analysis revealed a notable association between differentially expressed genes (DEGs) and the growth factor receptor interaction signaling pathway. Genes from *Gallus gallus* were used as the reference genome for KEGG annotation. The KEGG pathway maps highlight pathways associated with differential expression of ACTA1, MYBPC1, TGFβ2, IGF2, and MYH9. In addition, GO analysis further categorized these genes into Molecular Function (MF), Cellular Component (CC), and Biological Process (BP) terms related to muscle growth and cellular organization. Collectively, the KEGG and GO enrichment results indicate that genes associated with muscle development and growth‐related signaling pathways are transcriptionally active and co‐enriched with genes potentially involved in meat quality–related traits, as reflected by changes in mRNA expression of key growth‐associated genes.

FIGURE 1(a) Gene ontology (GO), molecular function (MF), cellular component (CC), biological process (BP)–gene count–fold enrichment, (b) KEGG–gene count–fold enrichment, (c) enriched pathways based on 472 DEGs. KEGG: Kyoto Encyclopedia of Genes and Genomes enrichment analysis results of muscle‐specific highly expressed genes.

**Figure:**
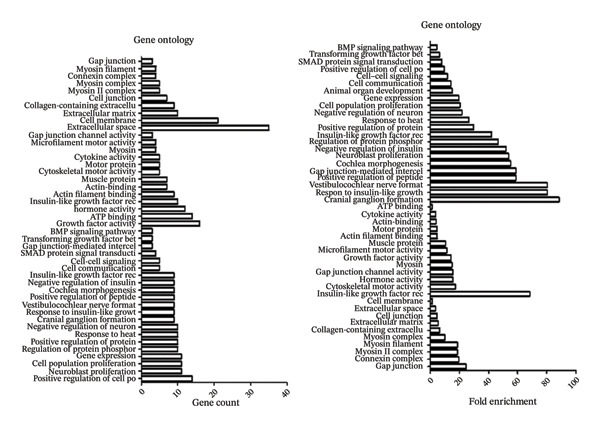
(a)

**Figure:**
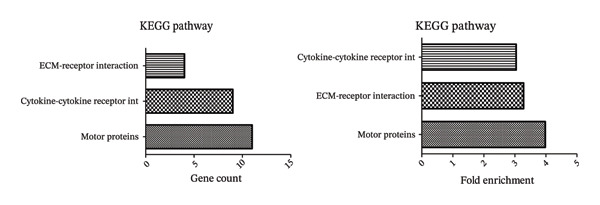
(b)

**Figure:**
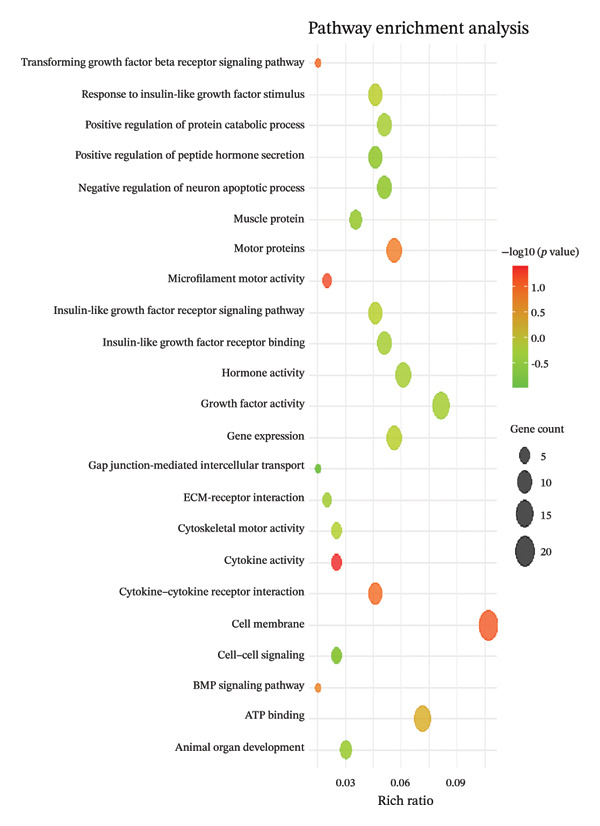
(c)

### 3.2. Gene Expression of Muscle Development

**FIGURE 2:**
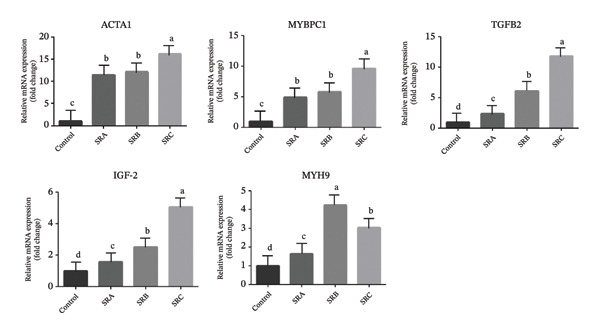
Effect of multienzyme on breast muscle mRNA expression of KUB chickens at ACTA1, MYBPC1, TGF‐β2, IGF2, and MYH9 genes “a–d” means within the same segment differ (*p* < 0.10, *p* = 0.01, and *p* < 0.05, respectively).

Significant differences in the expression patterns of five genes were detected in the breast muscle of KUB chickens. Across the multienzyme‐supplemented dietary treatments, the differential expression trends of ACTA1, MYBPC1, TGFβ2, IGF2, and MYH9 were generally consistent with the transcriptome sequencing results (Figure 1). It should be noted that these genes primarily represent structural or growth‐associated markers and changes in their mRNA expression reflect transcriptional responses rather than direct causal evidence of muscle fiber hyperplasia, meat quality improvement, or activation of specific signaling pathways. The mRNA expression of the ACTA1 gene in breast muscle tissue differed among multienzyme treatments (*p* < 0.05). Chickens receiving diets supplemented with 200 mg phytase and 300 mg protease per kg of feed exhibited higher ACTA1 mRNA expression compared with the control group without multienzyme supplementation; however, this difference was not significant when compared with the treatment containing 200 mg phytase and 500 mg protease (*p* > 0.05). Overall, ACTA1 expression showed a gradual increasing trend with increasing multienzyme dosage, although these differences were not statistically significant, suggesting an adaptive transcriptional response to improved nutrient utilization rather than a direct enhancement of muscle structural development. MYBPC1 gene expression differed significantly among treatments (*p* < 0.05). As shown in Figure 2, chickens fed diets supplemented with 200 mg phytase and 300 mg protease exhibited significantly higher MYBPC1 mRNA expression than the control group (*p* < 0.05), but no significant difference was observed when compared with the treatment containing 200 mg phytase and 500 mg protease (*p* > 0.05). The highest MYBPC1 expression level was observed in the group receiving 200 mg phytase and 700 mg protease, which was significantly higher than that in all other treatments (*p* < 0.05). These expression patterns likely indicate modulation of muscle‐related gene expression in response to dietary enzyme supplementation, rather than definitive evidence of increased muscle fiber number or improved meat quality.

TGFβ2 mRNA expression also differed significantly among dietary treatments (*p* < 0.05). Supplementation with 200 mg phytase and 300 mg protease did not result in a significant difference compared with the control group (*p* > 0.05). In contrast, the highest and most significant increase in TGFβ2 expression was observed in the treatment supplemented with 200 mg phytase and 700 mg protease (*p* < 0.05), which may reflect altered regulation of growth‐related signaling processes at the transcriptional level rather than direct activation of downstream signaling pathways. The mRNA expression of IGF2 in the breast muscle of male KUB chickens differed significantly among treatments with different multienzyme doses (*p* < 0.05). IGF2 expression increased progressively with increasing multienzyme supplementation, with significant upregulation observed in diets containing 200 mg phytase combined with 500 mg or 700 mg protease (*p* < 0.05). The highest IGF2 mRNA expression was detected in chickens fed diets supplemented with 200 mg phytase and 700 mg protease per kg of feed, which was significantly higher than that in all other treatment groups (Figure 2). However, given that IGF2 functions as a growth‐associated factor, these transcriptional changes should be interpreted as indicative of enhanced growth‐related gene expression rather than direct evidence of muscle accretion. Similarly, MYH9 mRNA expression differed significantly among multienzyme treatments (*p* < 0.05). The highest MYH9 expression level was observed in chickens receiving diets supplemented with 200 mg phytase and 500 mg protease, whereas further increases in multienzyme dosage resulted in a reduction in MYH9 mRNA expression in breast muscle tissue. Considering that MYH9 encodes a nonmuscle myosin involved primarily in cytoskeletal organization and cellular remodeling, this nonlinear expression pattern likely reflects differential regulation of cytoskeletal dynamics in response to varying nutrient availability, rather than a direct association with meat quality traits.

### 3.3. Effects on Performance and Carcass Characteristics

The effect of different multienzyme concentrations in feed on the performance of KUB chickens at different ages is presented in Table 2. At 15 days of age, chickens in the SRC group exhibited significantly lower cumulative FI, both overall and during the starter phase, compared with the control and other multienzyme‐treated groups (*p* < 0.05). In contrast, cumulative FI increased significantly (*p* < 0.05) in the control, SRA, and SRB groups at 30 and 45 days of age during the finisher phase (Table 2). Cumulative body weight differed significantly among treatments and ages. During the finisher and overall experimental periods, cumulative body weight in the 15‐day‐old control group remained significantly lower (*p* < 0.05) than that in all multienzyme‐treated groups (Table 2). It was seen that during the treatment period, cumulative body weight (g) was significantly higher (*p* < 0.05) in 45‐day SRC compared to the control and SRA at all ages, while that of 30‐day SRB remained comparable (Table 2). The cumulative body weight (g) of the 15‐day‐old control group remained significantly lower (*p* < 0.05) than that of SRA, SRB, and SRC at all ages (Table 2). It was observed that throughout the experimental, finisher, and overall experimental periods, the cumulative body weight remained significantly higher (*p* < 0.05) at 45 days of age across all treatments compared to the 15‐day‐old and 30‐day‐old groups with and without multienzymes in the feed at all ages. FCR was significantly reduced (*p* < 0.05) in the 45‐day‐old SRA and SRB groups compared with the control and all 15‐day‐old and 30‐day‐old groups (Table 2). Conversely, the highest FCR values were observed in the 15‐day‐old control group, followed by the 15‐day‐old SRA group. No significant differences in FCR were observed among the 30‐day‐old SRB and SRC groups or among the 45‐day‐old treatment groups during the finisher period.

**TABLE 2:** Production parameters feed intake, body weight, body weight gain, and feed conversion ratio.

Production parameter	Feed intake (g)			Body weight (g)			Body weight gain (g/chicken)			Feed conversion ratio (g feed: g weight again)		
Days	15	30	45	15	30	45	15	30	45	15	30	45
*Diets*												
Con	96.20 ± 3.23	207.50^c^ ± 3.32	319.00^c^ ± 4.97	172.55 ± 5.43	520.45^c^ ± 1.02	770.24^c^ ± 1.36	126.54 ± 0.57	405.06^c^ ± 1.49	541.37^c^ ± 0.69	3.47 ± 1.97	2.95^b^ ± 0.53	2.82^b^ ± 0.51
SRA	95.76 ± 2.41	205.00^b^ ± 2.16	316.75^b^ ± 3.50	194.75 ± 0.62	571.76^a^ ± 1.25	812.10^b^ ± 1.74	144.17 ± 0.65	414.04^b^ ± 0.82	536.32^b^ ± 1.39	3.17 ± 0.47	1.89^a^ ± 0.06	1.52^a^ ± 0.58
SRB	95.16 ± 1.68	203.25^a^ ± 6.40	309.50^a^ ± 4.04	194.62 ± 059	531.46^b^ ± 1.10	816.11^b^ ± 1.36	152.04 ± 0.97	424.33^a^ ± 1.12	540.46^b^ ± 0.53	2.84 ± 0.42	1.71^a^ ± 0.50	1.65^a^ ± 0.59
SRC	86.22 ± 7.71	202.25^a^ ± 4.57	308.25^a^ ± 1.50	195.63 ± 0.70	593.05^a^ ± 0.97	841.22^a^ ± 1.17	154.55 ± 0.52	446.51^a^ ± 0.54	674.97^a^ ± 0.74	2.80 ± 0.95	1.87^a^ ± 1.07	1.54^a^ ± 0.13
Diet effect	NS	0.003	0.005	NS	0.003	0.007	NS	0.004	0.002	NS	0.003	0.007

*Contrasts*												
Con vs. multienzyme	NS	0.005	0.005	NS	0.006	0.003	NS	0.004	0.003	NS	0.003	0.005

*Note:* ^a–c^Means with varying superscripts differ significantly (*p* < 0.05). Diets: Con: control (without phytase enzyme and protease enzyme), SRA: phytase enzyme at 200 mg/kg + protease enzyme at 300 mg/kg, SRB: phytase enzyme at 200 mg/kg + protease enzyme at 500 mg/kg, and SRC: phytase enzyme at 200 mg/kg + protease enzyme at 700 mg/kg.

The effects of dietary multienzyme supplementation on carcass characteristics are presented in Table 3. The control and various multienzyme groups showed comparable outcomes for the carcass and its various sections, including the breast, thighs, and wings (Table 3). Carcass percentage increased significantly (*p* < 0.05) in the SRB treatment compared to the other two multienzyme concentrations (SRA and control) but increased again at the higher multienzyme concentration (SRC) (Table 3). The results of the analysis showed that breast weight increased significantly (*p* < 0.05) in SRC when compared to the control but was not significantly different (*p* > 0.05) with the SRB treatment; furthermore, there was no significant difference (*p* > 0.05) in breast percentage. No significant effects were shown on body weight and abdominal fat weight, indicating that there was no response to increasing the calorie–protein ratio when multienzymes were added. Furthermore, body weight and abdominal fat percentage increased significantly (*p* < 0.05) in the multienzyme treatment when compared to the control. The percentage of abdominal fat decreased in both SRB and SRC treatments, as the multienzyme dose in the diet increased. The effects of multienzyme supplementation on organ development are shown in Table 4. The absolute weights of the liver, heart, gizzard, and spleen did not differ significantly among treatments (*p* > 0.05). In contrast, the weights of the thymus and bursa of Fabricius differed significantly among groups (*p* < 0.05). The relative weights of the liver and gizzard were significantly higher (*p* < 0.05) in the SRC group compared with the control, whereas heart percentage did not differ significantly between multienzyme‐supplemented and nonsupplemented groups. The percentages of the thymus and bursa of Fabricius were significantly higher (*p* < 0.05) in the SRC group than those of the SRA and SRB groups, while spleen percentage was significantly higher (*p* < 0.05) in the control group compared with all multienzyme‐treated groups (Table 4).

**TABLE 3:** Carcass characteristics (whole carcass, breast, thighs, and wings).

Organ	Carcass		Breast		Thighs		Abdominal fat	
	(g)	DP (%)	(g)	(%)	(g)	(%)	(g)	(%)
*Diets*								
Con	850 ± 32.61	74.39 ± 1.24	219.00^c^ ± 11.52	19.18 ± 1.05	241.75 ± 18.71	21.17^b^ ± 1.67	25.25 ± 0.62	2.21 ± 0.10
SRA	1164 ± 91.08	74.73 ± 1.42	258.25^b^ ± 12.18	16.63 ± 0.91	301.25 ± 15.41	19.39^c^ ± 1.03	23.57 ± 1.42	1.52 ± 0.12
SRB	1183 ± 40.51	76.29 ± 1.58	304.75^a^ ± 19.00	19.67 ± 1.24	324.25 ± 8.30	20.93^b^ ± 0.83	22.44 ± 0.40	1.50 ± 0.06
SRC	1215 ± 31.89	81.18 ± 1.83	321.25^a^ ± 11.59	21.46 ± 0.65	360.25 ± 5.97	24.08^a^ ± 1.10	21.94 ± 0.35	1.46 ± 0.03
Diet effect	NS	NS	0.04	NS	NS	0.03	NS	NS

*Contrasts*								
Con vs. multienzyme	0.05	0.02	0.04	NS	0.04	0.06	0.04	0.03

*Note:* ^a-c^Means with varying superscripts differ significantly (*p* < 0.05). DP: dressing %. Diets: Con: control (without phytase enzyme and protease enzyme), SRA: phytase enzyme at 200 mg/kg + protease enzyme at 300 mg/kg, SRB: phytase enzyme at 200 mg/kg + protease enzyme at 500 mg/kg, and SRC: phytase enzyme at 200 mg/kg + protease enzyme at 700 mg/kg.

**TABLE 4:** Organ weight and percent liver, heart, gizzard, thymus, lien, and bursa.

Organ	Liver		Heart		Gizzard		Thymus		Lien		Bursa Fabricius	
	(g)	(%)	(g)	(%)	(g)	(%)	(g)	(%)	(g)	(%)	(g)	(%)
*Diets*												
Con	25.80 ± 6.28	18.93 ± 2.61	6.88 ± 0.29	5.13 ± 0.72	29.88 ± 5.51	22.51 ± 6.11	5.57 ± 1.49	4.23 ± 1.62	3.50 ± 0.91	2.66 ± 1.02	2.57 ± 0.57	1.91 ± 0.42
SRA	42.31 ± 1.32	21.97 ± 1.81	8.88 ± 1.80	4.58 ± 0.80	28.40 ± 2.27	14.75 ± 1.72	11.58 ± 2.06	6.00 ± 1.08	4.48 ± 0.28	2.33 ± 0.26	4.95 ± 1.32	2.56 ± 0.63
SRB	43.77 ± 3.49	22.44 ± 2.76	10.27 ± 2.14	5.43 ± 0.43	27.45 ± 6.90	15.12 ± 2.15	14.17 ± 1.47	7.42 ± 0.94	4.60 ± 1.48	2.37 ± 0.34	5.10 ± 1.37	2.67 ± 0.52
SRC	46.38 ± 2.36	23.08 ± 1.35	10.51 ± 1.15	5.25 ± 0.73	26.82 ± 4.24	13.35 ± 2.23	14.67 ± 1.89	7.33 ± 1.22	4.80 ± 0.67	2.38 ± 0.27	6.20 ± 0.34	3.08 ± 0.21
Diet effect	NS	0.03	NS	NS	NS	0.04	0.02	0.03	NS	0.08	0.04	0.03

*Contrasts*												
Con vs. multienzyme	NS	0.06	NS	NS	NS	0.01	0.02	0.07	NS	0.03	0.03	0.05

*Note:*​ Organ% = weight of organ (g)/carcass weight (g). Diets: Con: control (without phytase enzyme and protease enzyme), SRA: phytase enzyme at 200 mg/kg + protease enzyme at 300 mg/kg, SRB: phytase enzyme at 200 mg/kg + protease enzyme at 500 mg/kg, SRC: phytase enzyme at 200 mg/kg + protease enzyme at 700 mg/kg.

### 3.4. Meat Quality Evaluation

Dietary multienzyme supplementation influenced several meat quality traits, including pH, texture, CL, WHC, and color parameters (Table 5). Meat quality analysis indicated that pH and texture were not significantly affected by multienzyme supplementation compared with the control diet (*p* > 0.05). In contrast, significant differences were observed in CL and WHC between chickens fed multienzyme‐supplemented diets and those fed the control diet (*p* < 0.05), as presented in Table 5. Meat color parameters were significantly affected by dietary multienzyme supplementation (*p* < 0.05). Lightness (*L* ^∗^) values differed significantly among treatments, with notable differences observed between lower (SRA) and higher multienzyme inclusion levels (SRB and SRC). Redness (*a* ^∗^) of KUB chicken meat was significantly higher in all multienzyme‐treated groups (SRA, SRB, and SRC) compared with the control group (SR) (*p* < 0.05). Conversely, yellowness (*b* ^∗^) values showed a significant decrease in the SRB and SRC groups compared with the SRA group (*p* < 0.05). Moreover, the highest multienzyme inclusion level (SRC) resulted in a significantly lower *b* ^∗^ value than that observed in the control group without multienzyme supplementation (*p* < 0.05).

**TABLE 5:** Meat quality traits pH, cooking loss, water‐holding capacity, shear force, and color attributes (*L* ^∗^, *a* ^∗^, and *b* ^∗^).

Quality trait	pH	Texture (N)	CL (%)	WHC (%)	*L*	*a*	*b*
*Diets*							
Con	5.87 ± 0.45	19.37 + 2.97	35.58 + 0.78	21.31 + 5.85	43.73 + 2.39	4.13^d^ + 1.72	14.15^d^ + 1.40
SRA	5.81 ± 0.19	21.24 + 1.06	29.65 + 5.51	28.63 + 0.51	48.67 + 2.51	7.77^c^ + 2.01	10.28^c^ + 0.68
SRB	5.98 ± 0.16	21.31 + 1.02	29.51 + 3.90	28.78 + 1.59	49.89 + 1.57	8.73^b^ + 0.70	9.45^b^ + 1.01
SRC	5.79 ± 0.18	21.48 + 0.85	28.65 + 2.46	29.98 + 1.10	50.61 + 3.68	9.06^a^ + 0.49	8.49^a^ + 1.22
Diet effect	NS	NS	NS	0.02	NS	0.02	0.03

*Contrasts*							
Con vs. multienzyme	NS	NS	0.05	0.03	0.02	0.03	0.05

*Note: L*, lightness; *a*, redness; *b*, yellowness. Diets: Con: control (without phytase enzyme and protease enzyme), SRA, phytase enzyme at 200 mg/kg + protease enzyme at 300 mg/kg; SRB, phytase enzyme at 200 mg/kg + protease enzyme at 500 mg/kg; SRC, and phytase enzyme at 200 mg/kg + protease enzyme at 700 mg/kg.

Abbreviations: CL, cooking loss; WHC, water‐holding capacity.

## 4. Discussion

### 4.1. Novel Application of Multienzyme on Muscle Composition

Meat quality exhibits substantial variability both within and among chicken strains as a result of interacting genetic, nutritional, and environmental influences. Meat quality is greatly influenced by sensory qualities, flavor, and softness [22]. The ACTA1 gene has been implicated as an important molecular contributor to muscle structure, consequently, meat quality traits [23]. It is desirable for customers and the chicken industry to increase the levels of other genes, including MYBPC1 and MYH9, and is desirable to enhance meat quality while maintaining overall carcass yield [24, 25]. Moreover, TGFβ2 and IGF2 have been reported to contribute to meat quality improvement, although their roles are primarily associated with growth regulation and muscle development rather than direct modulation of dietary nutrient composition [26, 27]. In this study, breast muscles of KUB chickens fed with different multienzyme doses in the feed were compared and analyzed. Transcriptome analysis identified a set of DEGs with potential relevance to muscle development and meat quality traits. Validation of selected DEGs using quantitative real‐time PCR (qRT‐PCR) confirmed the reliability of the transcriptomic data. Functional enrichment analysis further revealed significant enrichment of receptor interaction–related signaling pathways associated with muscle growth and meat quality, as indicated by KEGG pathway analysis (Figure 1).

### 4.2. Gene Expression of Muscle Tissue

The ACTA1, MYBPC1, TGFβ2, IGF2, and MYH9 genes are mediate biological mechanisms that contribute to improvements in both meat quality and overall performance in chickens. Numerous meat quality–related genes are concurrently enriched within the multigene receptor interaction signaling pathway, as reflected by their mRNA expression profiles. TGFβ2 and IGF2 genes are mediate mechanisms involved in improving meat quality and performance in chickens. Numerous meat quality–related genes are concurrently enriched within the multigene receptor interaction signaling pathway, as reflected by their mRNA expression profiles [28]. Based on the research data, the results of the gene expression images were quite interesting. Because in general, the administration of a combination of phytase enzyme and protease enzyme to KUB chicken exposed to the gene expression of ACTA1, MYBPC1, TGFβ2, IGF2, and MYH9. Overall, multienzyme inclusion in the diet resulted in an upregulation of these genes, as illustrated in Figure 2. The estimated genetic parameters for traits associated with the markers showed additive genetic variability large enough to be used in selection, indicating the importance of finding genetic relationships of molecular markers with growth‐promoting traits in chickens [29, 30]. The identification and utilization of quantitative trait loci (QTLs) can accelerate genetic progress in poultry breeding programs [31], while gene expression profiles and myogenic regulatory factors governing muscle development provide valuable insights for enhancing poultry meat production [32]. The observed genetic relationship with performance between ACTA1 and MYBPC1 is highly desirable [33, 34]. The MYBPC1 gene plays a pivotal role in muscle fiber development and intramuscular fat deposition [34]. Furthermore, genetic factors play a key role in determining body weight at different stages of growth, with IGF2 being particularly influential and MYH9, which supports muscle cell proliferation and protein autophosphorylation exhibiting strong positive genetic correlations with improved meat yield and carcass quality in broiler breeding programs [35, 36]. The nature of increasing meat quality and muscle mass is genetically associated with growth genes and other molecular markers [37]. Among these, ACTA1 serves as a critical molecular marker for detecting enhanced muscle development and increased muscle fiber formation in chickens [38], whereas TGFβ2 contributes to improved meat composition and overall meat quality [39].

### 4.3. Enhanced Performance and Carcass Potency of Multienzyme

The performance outcomes of KUB chickens receiving different multienzyme doses suggest that enhanced digestibility in the digestive tract improves the chicken’s capacity to extract nutrients that would otherwise remain limited [38]. Feed intake decreased at the addition of multienzyme levels with different doses. The lowest feed consumption was 308.25 g in the SRC treatment of 45‐day‐old chickens, when compared to controls at the same age. As KUB chickens age, their digestive ability increases which is supported by potential phytochemicals in the feed and the type of feed given [39]. The reduction in FI associated with multienzyme supplementation may reflect the ability of chickens to meet their nutritional requirements with lower feed consumption due to improved energy and amino acid digestibility, rather than increased total nutrient intake [40]. In the 30‐day‐old group, FI was relatively similar among treatments, with values of 205.00 g (SRA), 203.25 g (SRB), and 202.25 g (SRC) and showed only minor differences compared with the 15‐day‐old group. Another explanation for the lack of reaction could be that the chickens were fed a high‐quality food that enabled them to function nearly at their genetic capacity [41]. Final body weight increased with increasing multienzyme inclusion levels, with the 45‐day‐old SRC group exhibiting higher body weight compared with other treatments and the control group. Improvements in growth performance are likely influenced by the formulation and specificity of the multienzyme mixture, as the degradation of antinutritional factors such as phytate may be enhanced through enzymatic action [42]. Consequently, there was no significant effect on the FCR in the 15‐day‐old group of chickens, which means that multienzymes are not able to modify the digestive environment to increase feed utilization efficiency. In contrast, FCR decreased at 30 and 45 days of age with multienzyme supplementation, indicating improved feed efficiency at later growth stages. Variations in FCR may also be influenced by genetic background, diet composition, and housing or management systems [43].

### 4.4. Effect on Digestive Organ Development

Improved feed efficiency has been associated with the development of digestive organ morphology, and previous studies have consistently reported that chickens with higher feed efficiency tend to exhibit a more developed gizzard [44]. The gizzard serves as a primary site for the mechanical processing of feed in poultry [45]. A robust muscle layer’s shrinking is generally linked to a well‐developed gizzard, which can improve feed efficiency [46]. These results imply that increasing the effectiveness of chicken feed may depend on the organ’s extrusion and trituration processes [47]. In chickens, the liver represents the largest glandular organ of the digestive system and plays a critical role in nutrient metabolism through bile synthesis, which facilitates lipid digestion by activating pancreatic lipase [48]. These findings are in line with earlier studies on the digestive systems of broiler chicks that were 23 days old and had good feed efficiency [49]. Low feed efficiency chicken livers can make up for the lack of gizzard digestion by enhancing small intestine absorption and digestion [50]. Additionally, discovered in this study that multienzymes reduced the concentration of belly fat in broiler chicks and raised liver weight. One popular technique for assessing a chicken’s immunological state is to weigh its immune organs [51]. These organs include the liver, heart, gizzard, thymus, spleen, and bursa of Fabricius, all of which play essential roles in maintaining optimal health and performance [52]. The observed increase in liver weight in multienzyme‐supplemented chickens may reflect enhanced metabolic activity and improved nutrient utilization compared with the control group. However, it should be noted that increased hepatic lipid deposition may also contribute to greater liver weight [53]. Although multienzymes may influence feed efficiency through multiple physiological and biochemical mechanisms, including enhanced enzymatic activity, this does not diminish the fundamental role of digestive organ development in determining overall feed utilization efficiency.

### 4.5. Effect of Meat Quality in KUB Chicken

The effect of adding multienzymes to feed on pH, texture, CL, WHC, and also meat color such as lightness (*L* ^∗^), redness (*a* ^∗^), and yellowness (*b* ^∗^) is shown in Table 5. The pH level is an important parameter in meat quality because pH can affect CL and meat texture [54]. In general, meat lightness (*L* ^∗^) and yellowness (*b* ^∗^) are negatively correlated with pH, whereas redness (*a* ^∗^) shows a positive correlation with pH (Table 5). Analysis of breast muscle samples from chickens fed multienzyme‐supplemented diets revealed a reduction in yellowness (*b* ^∗^) and an increase in redness (*a* ^∗^) values. The correlation coefficients between color parameters (*L* ^∗^, *a* ^∗^, and *b* ^∗^) and pH in multienzyme‐treated samples were highly significant, indicating coordinated changes in physicochemical properties. Physical and biochemical alterations in meat, including microbial contamination such as Salmonella, may contribute to reductions in pH and deterioration of texture [55]. Previous observations have also shown that lower pH values accompanied by higher redness (*a* ^∗^) can influence overall meat quality, with high‐quality chicken meat often characterized by increased lightness (*L* ^∗^) and redness (*a* ^∗^) [56].

Cooking loss decreased significantly with increasing levels of multienzyme supplementation, concomitant with improvements in meat texture and WHC (Table 5). According to previous studies, the addition of multienzymes in the feed can contribute to its high WHC [57]. Good WHC contributed by multienzymes in the feed resulted in lower CL in meat. The weight difference between the raw and cooked samples, represented as a percentage of the initial weight, was used to compute CL [58]. The SRC sample showed the highest WHC because multienzymes in the feed can reduce CL due to their capacity to retain water [59]. This outcome could be the consequence of the feed’s increased multienzyme content, which increases the meat’s ability to retain water and forms a compact, hard structure [60]. Furthermore, both the type and dosage of multienzymes appeared to influence meat texture. Enzymatic and biochemical modifications in muscle tissue can alter meat color and texture, traits that are frequently associated with improved meat quality [61]. Correlation analysis indicated that multienzyme supplementation improved textural properties synergistically, while higher CL was significantly associated with increased chewiness and firmness, reflecting a tougher meat texture [62].

## 5. Conclusion

The provision of multienzymes (a combination of phytase and protease) in the feed of KUB chicken has been shown to improve growth performance, feed efficiency, and modulate the expression of genes related to growth and muscle development. Multienzyme supplementation, especially at higher doses, is associated with increased final body weight, improved feed conversion in the final phase of rearing, and positive changes in carcass characteristics and digestive organs. Furthermore, multienzymes affect meat quality by increasing WHC, reducing CL, and improving meat color parameters. Changes in the expression of ACTA1, MYBPC1, TGFβ2, IGF2, and MYH9 genes indicate a transcriptional response to increased nutrient utilization, potentially supporting growth and meat quality. Overall, multienzyme supplementation is an effective and prospective nutritional strategy to improve the productivity and meat quality of KUB chickens.

## Funding

This research was supported by the Indonesian Education Scholarship (BPI), Center for Higher Education Funding and Assessment (PPAPT), and Indonesian Endowment Fund for Education (LPDP), Ministry of Higher Education, Science and Technology of Republik Indonesia, grant number: 01366/BPPT/BPI.06/9/2023.

## Ethics Statement

The research protocol was reviewed and approved by the Animal Care and Use Committee of the Faculty of Veterinary Medicine, Universitas Airlangga (Approval No: 1.KEH.038.03.2024).

## Conflicts of Interest

The authors declare no conflicts of interest.

## Data Availability

The data supporting this study are not publicly available due to privacy or ethical restrictions.

## References

[1] Qi L. , Xiao L. , Fu R. , Nie Q. , Zhang X. , and Luo W. , Genetic Characteristics and Selection Signatures Between Southern Chinese Local and Commercial Chickens, Poultry Science. (2024) 103, no. 7, 10.1016/j.psj.2024.103863. PMC11166977 38810566

[2] Ayuti S. R. , Shin S. , Kim E. J. et al., ACTA1 Gene Regulation in Livestock: A Multidimensional Review on Muscle Development, Meat Quality, and Genetic Applications, Veterinary World. (2025) 18, no. 8, 2520–2541, 10.14202/vetworld.2025.2520-2541. 41064813 PMC12501589

[3] Erwan E. , Adelina T. , Koto A. , and Maslami V. , The Potency of Oral Administration of L-Citrulline as Anti Heat Stress Agent in KUB Chicks, Journal of World’s Poultry Science. (2020) 10, no. 1, 36–40, 10.36380/jwpr.2020.5.

[4] Sinurat A. P. , Haryati T. , Herliatika A. , and Pratiwi N. , Performances of KUB Chickens Fed Diets With Different Nutrient Densities and BS4 Enzyme Supplementation, Tropical Animal Science Journal. (2022) 45, no. 1, 73–83, 10.5398/tasj.2022.45.1.73.

[5] Hidayah R. , Ambarsari I. , and Subiharta S. , Study of Physical, Nutritional and Sensory Properties KUB Chicken Meat in Central Java, Indonesian Journal of Animal Science. (2019) 21, no. 2, 93–101, 10.25077/jpi.21.2.93-101.2019.

[6] Ayuti S. R. , Khairullah A. R. , Lamid M. et al., Avian Influenza in Birds: Insights From a Comprehensive Review, Veterinary World. (2024) 17, no. 11, 2544–2555, 10.14202/vetworld.2024.2544-2555. 39829652 PMC11736375

[7] Muhammad A. , Sultan A. , Khan S. , and Sadique U. , Effect of Phytase, Protease, and Their Combination on the Nutrients Bioavailability and Performance Indices of Broilers Fed a Sorghum-Based Diet Under Local Climatic Conditions of Khyber Pakhtunkhwa Pakistan, Pakistan Journal of Zoology. (2025) 57, no. 1, 167–173, 10.17582/journal.pjz/20230302130319.

[8] Amiri M. Y. A. , Jafari M. A. , and Irani M. , Growth Performance, Internal Organ Traits, Intestinal Morphology, and Microbial Population of Broiler Chickens Fed Quinoa Seed-Based Diets With Phytase or Protease Supplements and Their Combination, Tropical Animal Health and Production. (2021) 53, no. 6, 10.1007/s11250-021-02980-0. 34743230

[9] Ahmadi M. , Ghasemi H. A. , Hajkhodadadi I. , and Khaligh F. , Effect of an *Escherichia coli*-Derived Phytase and a Carbohydrase-Protease Cocktail Derived From *Bacillus* Spp. on Performance, Digestibility, Bone Mineralization and Gut Morphology in Broilers Fed Different Nutrient Density Diets, Veterinary Medicine and Science. (2024) 10, no. 1, 10.1002/vms3.1344. PMC10790325 38227704

[10] Abd El Latif M. A. , Abdel-Wareth A. A. A. , Daley M. , and Lohakare J. , Effect of Dietary Orange Peel Meal and Multi-Enzymes on Productive, Physiological and Nutritional Responses of Broiler Chickens, Animals. (2023) 13, no. 15, 10.3390/ani13152473. PMC10416891 37570281

[11] İpçak H. H. , Cardozo P. W. , Denli M. , and Escobero S. J. , Effect of Multi-Enzyme Complex and Feed Form on Growth Performance, Slaughter Characteristic, Total Tract Nutrient Digestibility, and Energy Utilization in Broiler Chickens, Journal of Animal Science and Veterinary Medicine. (2022) 7, no. 6, 198–204, 10.31248/JASVM2022.354.

[12] Muhsinin M. , Maskur M. , Jan R. , Rozi T. , Kasip L. M. , and Al Farizi M. S. , Identification of Diversity and Genetic Distance of Indonesian Local Chicken Strains Based on Myostatin Gene, Jurnal Biologi Tropis. (2025) 25, no. 1, 18–25, 10.29303/jbt.v25i1.8297.

[13] Ayuti S. R. , Lamid M. , Warsito S. H. et al., A Review of Myostatin Gene Mutations: Enhancing Meat Production and Potential in Livestock Genetic Selection, Open Veterinary Journal. (2024) 14, no. 12, 3189–3202, 10.5455/OVJ.2024.v14.i12.4. 39927343 PMC11799654

[14] Lei Q. , Zhang S. , Wang J. et al., Genome-Wide Association Studies of Egg Production Traits by Whole Genome Sequencing of Laiwu Black Chicken, Poultry Science. (2024) 103, no. 6, 10.1016/j.psj.2024.103705. PMC11636908 38598913

[15] Chen L. , Wang X. , Cheng D. et al., Population Genetic Analyses of Seven Chinese Indigenous Chicken Breeds in a Context of Global Breeds, Animal Genetics. (2019) 50, no. 1, 82–86, 10.1111/age.12732, 2-s2.0-85056429869. 30421435

[16] Saragih H. T. S. S. G. , Salsabila N. , Deliaputri R. , Firdaus A. B. I. , and Kurnianto H. , Growth Morphology of the Gastrointestinal Tract, Pectoralis Thoracicus Muscle, Lymphoid Organ and Visceral Index of Kampong Chicken, Journal of the Saudi Society of Agricultural Sciences. (2024) 23, no. 1, 34–41, 10.1016/j.jssas.2023.08.005.

[17] Warsito S. H. , Lamid M. , Al-Arif M. A. et al., The Results of Intestinal Villi of Laying Hens Exposed With Avian Pathogenic *Escherichia coli* (APEC) After Giving Citric Acid and Dextrose, Veterinary Medicine International. (2025) 2025, 6623764–8, 10.1155/vmi/6623764. 40041134 PMC11876522

[18] Zhu H. , Li X. , Wang J. et al., Transcriptomic Analysis Reveals Differentially Expressed Genes Associated With Meat Quality in Chinese Dagu Chicken and AA+ Broiler Roosters, BMC Genomics. (2024) 25, no. 1, 10.1186/s12864-024-10927-6. PMC11515088 39455924

[19] Yang K. , Zhang J. , Zhao Y. et al., Whole Genome Resequencing Revealed the Genetic Relationship and Selected Regions Among Baicheng-You, Beijing-You, and European-Origin Broilers, Biology. (2023) 12, no. 11, 10.3390/biology12111397. PMC10669838 37997996

[20] Yulianto A. B. , Lamid M. , Lokapirnasari W. P. et al., Hematological and Performance Variables of Male Broiler Chickens Fed With *Moringa oleifera* Extract and Probiotic in Drinking Water, Tropical Animal Science Journal. (2024) 47, no. 2, 215–224, 10.5398/tasj.2024.47.2.215.

[21] Roobab U. , Chen B. R. , Madni G. M. et al., Evaluation of Ultrasound and Pulsed Electric Field Combinations on the Cooking Losses, Texture Profile, and Taste-Related Amino Acids of Chicken Breast Meat, Ultrasonics Sonochemistry. (2024) 107, no. 1, 10.1016/j.ultsonch.2024.106919. PMC11144803 38795569

[22] Tan X. , Liu R. , Zhao D. et al., Large-Scale Genomic and Transcriptomic Analyses Elucidate the Genetic Basis of High Meat Yield in Chickens, Journal of Advanced Research. (2024) 55, no. 1, 1–16, 10.1016/j.jare.2023.02.016. 36871617 PMC10770282

[23] Venturini G. C. , Stafuzza N. B. , Cardoso D. F. et al., Association Between ACTA1 Candidate Gene and Performance, Organs and Carcass Traits in Broilers, Poultry Science. (2015) 94, no. 12, 2863–2869, 10.3382/ps/pev285, 2-s2.0-84960465185. 26476088

[24] Li J. , Chen C. , Zhao R. , Wu J. , and Li Z. , Transcriptome Analysis of mRNAs, lncRNAs, and miRNAs in the Skeletal Muscle of Tibetan Chickens at Different Developmental Stages, Frontiers in Physiology. (2023) 14, no. 1, 10.3389/fphys.2023.1225349. PMC10410567 37565148

[25] Jia Q. H. , Cao Y. Z. , Xing Y. X. et al., LncRNA lncLLM Facilitates Lipid Deposition by Promoting the Ubiquitination of MYH9 in Chicken LMH Cells, International Journal of Molecular Sciences. (2024) 25, no. 19, 10.3390/ijms251910316. PMC11477197 39408647

[26] Lu J. , Yuan H. , Liu S. et al., Gene Coexpression Network Analysis Reveals the Genes and Pathways in Pectoralis Major Muscle and Liver Associated With Wooden Breast in Broilers, Poultry Science. (2024) 103, no. 10, 10.1016/j.psj.2024.104056. PMC11342257 39094498

[27] Mariandayani H. N. , Darwati S. , Khaerunnisa I. , and Prasasty V. D. , Growth Performance of Indonesian Three-Breed Cross Chicken Associated With Growth Hormone and Insulin-Like Growth Factor 2 Genes, Veterinary World. (2023) 16, no. 12, 2471–2478, 10.14202/vetworld.2023.2471-2478. 38328357 PMC10844795

[28] San J. , Du Y. , Wu G. , Xu R. , Yang J. , and Hu J. , Transcriptome Analysis Identifies Signaling Pathways Related to Meat Quality in Broiler Chickens-The Extracellular Matrix (ECM) Receptor Interaction Signaling Pathway, Poultry Science. (2021) 100, no. 6, 10.1016/j.psj.2021.101135. PMC8105667 33940279

[29] Zhang S. , Zhang J. , Cao C. et al., Effects of Different Rearing Systems on Lueyang Black-Bone Chickens: Meat Quality, Amino Acid Composition, and Breast Muscle Transcriptome, Genes. (2022) 13, no. 10, 10.3390/genes13101898. PMC9601429 36292783

[30] Liu Z. , Liu Y. , Xing T. et al., Transcriptome Analysis Reveals the Mechanism of Chronic Heat Stress on Meat Quality of Broilers, Journal of Animal Science and Biotechnology. (2022) 13, no. 1, 10.1186/s40104-022-00759-3. PMC9484139 36117193

[31] Hajibarat Z. , Saidi A. , Zeinalabedini M. , Mardi M. , and Ghaffari M. R. , Integrating Quantitative Trait Loci (QTLs) Through Meta-QTL (MQTL) Analysis to Facilitate Identification of Relationships Between Phenotype and Genotype, Biology Bulletin of the Russian Academy of Sciences. (2024) 51, no. 1, 1761–1776, 10.1134/S1062359024606888.

[32] Choi S. , Park J. W. , Lee S. I. , and Shin S. , Overexpression of Syndecan-4 Inhibits Myogenesis by Regulating the Expression of Myogenic Regulatory Factors, Journal of Animal Science And Technology. (2025) 67, no. 2, 410–420, 10.5187/jast.2024.e8. 40264532 PMC12010220

[33] Clayton J. S. , Johari M. , Taylor R. L. et al., An Update on Reported Variants in the Skeletal Muscle α‐Actin (ACTA1) Gene, Human Mutation. (2024) 2024, 6496088–19, 10.1155/2024/6496088. 40225930 PMC11918651

[34] Xu Y. , Huang Y. , Wei S. et al., Changes in Gut Microbiota Affect DNA Methylation Levels and Development of Chicken Muscle Tissue, Poultry Science. (2025) 104, no. 3, 10.1016/j.psj.2025.104869. PMC11874558 39952142

[35] Suswoyo I. , Mugiyono S. , and Tugiyanti E. , Genetic Variability of IGF1 and IGF2 and Correlation to Body Weight in Kedu Chicken of Indonesia, Biodiversitas: Journal of Biological Diversity. (2024) 25, no. 12, p4547–p4852, 10.13057/biodiv/d251220.

[36] Wei C. , Niu Y. , Chen B. et al., Divergent Regulatory Roles of Transcriptional Variants of the Chicken LDB3 Gene in Muscle Shaping, Journal of Agricultural and Food Chemistry. (2024) 72, no. 21, 12240–12250, 10.1021/acs.jafc.4c00520. 38764183

[37] Kumaravel V. , Mohan B. , Natarajan A. , Murali N. , Selvaraj P. , and Vasanthakumar P. , Effect on Growth Performance, Carcass Traits, and Myostatin Gene Expression in Aseel Chicken Fed Varied Levels of Dietary Protein in Isocaloric Energy Diets, Tropical Animal Health and Production. (2023) 55, no. 1, 10.1007/s11250-023-03505-7. 36795279

[38] Piga D. , Rimoldi M. , Magri F. et al., Case Report: A Novel ACTA1 Variant in a Patient With Nemaline Rods and Increased Glycogen Deposition, Frontiers in Neurology. (2024) 15, no. 1, 10.3389/fneur.2024.1340693. PMC10944937 38500810

[39] Zhao J. , Chen M. , Luo Z. , Cui P. , Ren P. , and Wang Y. , Strand-Specific RNA Sequencing Reveals Gene Expression Patterns in F1 Chick Breast Muscle and Liver After Hatching, Animals. (2024) 14, no. 9, 10.3390/ani14091335. PMC11083249 38731340

[40] Jin D. , Tugiyanti E. , Rimbawanto E. A. et al., Effects of High-Level Dietary Distillers Dried Grains With Solubles Supplemented With Multienzymes on Growth Performance, Nutrient Utilization, Intestinal Morphology, and Pellet Quality in Broiler Chickens, Veterinary World. (2024) 17, no. 8, 1943–1954, 10.14202/vetworld.2024.1943-1954. 39328431 PMC11422655

[41] Al Arif M. A. , Warsito S. H. , Lamid M. et al., Phytochemical Analysis of Curry Leaf Extract (*Murraya koenigii* L.) as a Potential Animal Feed and Medicinal Ingredient, Pharmacognosy Journal. (2024) 16, no. 2, 471–477, 10.5530/pj.2024.16.75.

[42] Iwujia T. , Iheanachoa G. , Ogambaa M. , and Odunfab O. , Relationship Between Live Weight, Internal Organs, and Body Part Weights of Broiler Chickens, Malaysian Animal Husbandry Journal. (2022) 2, no. 2, 64–66, 10.26480/mahj.02.2022.64.66.

[43] He S. , Hao X. , Wang S. et al., Starch Synthase II Plays a Crucial Role in Starch Biosynthesis and the Formation of Multienzyme Complexes in Cassava Storage Roots, Journal of Experimental Botany. (2022) 73, no. 8, 2540–2557, 10.1093/jxb/erac022. 35134892

[44] Hashim M. , Gonzalez-Sanchez D. , Wealleans A. , Abdelkader M. , El-Safty S. A. R. , and Abdelhady A. R. Y. , Effects of Different Doses of Multienzyme Supplementation on Growth Performance, Duodenal pH and Morphology, and Carcass Traits in Broilers Fed Diets With an Increasing Reduction in Energy, Animals. (2023) 13, no. 14, 10.3390/ani13142378. PMC10376475 37508155

[45] Ayuti S. R. , Lamid M. , Warsito S. H. et al., Supplementation of Phytase and Protease Enzymes on the Performance of KUB Chicken Using the MSTN Gene Expression, Asian Journal of Dairy and Food Research. (2025) 44, 188–198, 10.18805/ajdfr.DRF-461.

[46] Rostampour B. , Chamani M. , Seidavi A. , Zarei A. , and Karimi N. , The Effect of *Froriepia subpinnata* on the Performance, Carcass Characteristics, Blood Parameters, Immune System, Microbial Population, Intestinal Morphology, and Breast Meat Fatty Acid Content of Broiler Chickens, Tropical Animal Health and Production. (2024) 56, no. 1, 10.1007/s11250-024-03887-2. 38217627

[47] Al-Ruwad S. H. , Attia A. I. , Monem U. M. A. et al., Dietary Supplementation With Copper Nanoparticles Enhances Broiler Performance by Improving Growth, Immunity, Digestive Enzymes, and Gut Microbiota, Poultry Science. (2024) 103, no. 10, 10.1016/j.psj.2024.104026. PMC11338117 39067121

[48] Azizi M. , Bouyeh M. , and Seidavi A. , Effects of Different Levels of Fenofibrate on Growth Performance, Carcase Characteristics, Abdominal Fat, Serum Constitutes, Immune System, Caeca and Microbial Flora of Broilers, Italian Journal of Animal Science. (2022) 21, no. 1, 343–349, 10.1080/1828051X.2022.2032417.

[49] Al-Arif M. A. , Lamid M. , Rimayanti R. et al., Lack of Effect of Xylanase and Cellulase Supplementation on Nutrient Utilization in KUB Chickens Fed a Low-NSP Diet, Advances in Animal and Veterinary Sciences. (2025) 13, no. 12, 2693–2701, 10.17582/journal.aavs/2025/13.12.2693.2701.

[50] Mishra P. , Das R. , Chaudhary A. , Mishra B. , and Jha R. , Effects of Microalgae, With or Without Xylanase Supplementation, on Growth Performance, Organs Development, and Gut Health Parameters of Broiler Chickens, Poultry Science. (2023) 102, no. 11, 10.1016/j.psj.2023.103056. PMC10518709 37722276

[51] Facey H. , Kithama M. , Mohammadigheisar M. , Huber L. A. , Shoveller A. K. , and Kiarie E. G. , Complete Replacement of Soybean Meal With Black Soldier Fly Larvae Meal in Feeding Program for Broiler Chickens From Placement Through to 49 Days of Age Reduced Growth Performance and Altered Organs Morphology, Poultry Science. (2023) 102, no. 1, 10.1016/j.psj.2022.102293. PMC9709236 36442308

[52] Ivarsson E. , Wattrang E. , Sun L. , Cervin G. , Pavia H. , and Wall H. , Evaluation of Early Feed Access and Algal Extract on Growth Performance, Organ Development, Gut Microbiota and Vaccine-Induced Antibody Responses in Broiler Chickens, Animal. (2022) 16, no. 5, 10.1016/j.animal.2022.100522. 35468509

[53] Ampode K. M. and Mendoza F. C. , Oregano (*Origanum vulgare* Linn.) Powder as Phytobiotic Feed Additives Improves the Growth Performance, Lymphoid Organs, and Economic Traits in Broiler Chickens, Advances in Animal and Veterinary Sciences. (2022) 10, no. 2, 434–441, 10.17582/journal.aavs/2022/10.2.434.441.

[54] Hao D. , Tu X. , Zhang X. et al., Effects of Proteases Inactivation on Textural Quality of Yellow-Feathered Chicken Meat and the Possible Mechanism Based on Myofibrillar Protein, Food Control. (2024) 166, no. 1, 10.1016/j.foodcont.2024.110713.

[55] Ayuti S. R. , Khairullah A. R. , Al-Arif M. A. et al., Tackling Salmonellosis: A Comprehensive Exploration of Risks Factors, Impacts, and Solutions, Open Veterinary Journal. (2024) 14, no. 6, 1313–1329, 10.5455/ovj.2024.v14.i6.1. 39055762 PMC11268913

[56] Kim S. Y. , Song D. H. , Chung W. , Choi H. S. , Han S. G. , and Kim H. W. , Evaluation of the Physicochemical Attributes of Beef, Chicken, and Pork Muscles Injected With Microbial Proteases for Designing Senior-Friendly Processed Meat Products, Foods. (2024) 13, no. 21, 10.3390/foods13213430. PMC11545073 39517214

[57] Islam M. , Huang Y. , Jain P. , Fan B. , Tong L. , and Wang F. , Enzymatic Hydrolysis of Soy Protein to High Moisture Textured Meat Analogue With Emphasis on Antioxidant Effects: As a Tool to Improve Techno-Functional Property, Biocatalysis and Agricultural Biotechnology. (2023) 50, no. 1, 10.1016/j.bcab.2023.102700.

[58] Yaqoob M. U. , Yousaf M. , Iftikhar M. et al., Effect of Multi-Enzymes Supplementation on Growth Performance, Meat Quality, Ileal Digestibility, Digestive Enzyme Activity and Caecal Microbiota in Broilers Fed Low-Metabolizable Energy Diet, Animal Bioscience. (2022) 35, no. 7, 1059–1068, 10.5713/ab.21.0402. 35073663 PMC9271380

[59] Lee S. , Jo K. , Jeong S. K. , Jeon H. , Choi Y. S. , and Jung S. , Recent Strategies for Improving the Quality of Meat Products, Journal of Animal Science And Technology. (2023) 65, no. 5, 895–911, 10.5187/jast.2023.e94. 37969348 PMC10640940

[60] Wang B. , Lu H. , Lou H. , Acharya D. R. , Shi Y. , and Chen Q. , Synthesis and Characterization of Neurospora Intermedia-Based Composite Mycoprotein Gel Meat: Insight Into the Effect of pH and Soluble Starch on Water-Holding Capacity and Texture Properties, Food Hydrocolloids. (2024) 155, no. 1, 10.1016/j.foodhyd.2024.110190.

[61] Soisuwan K. , Plaimast H. , Thongnum A. , Chotnipat S. , and Nopparatmaitree M. , Effect of Natural Multi-Enzyme Supplementation on Growth Performance, Gut Microflora, Carcass Characteristics, and Meat Quality of Broilers Reared in Tropical Climates, Journal of Animal Health and Production. (2023) 11, no. 2, 129–138, 10.17582/journal.jahp/2023/11.2.129.138.

[62] Attia Y. A. , Al-Khalaifah H. S. , Abdulmohsen H. A. et al., The Impact of Multi-Enzyme Fortification on Growth Performance, Intestinal Morphology, Nutrient Digestibility, and Meat Quality of Broiler Chickens Fed a Standard or Low-Density Diet, Frontiers in Veterinary Science. (2022) 9, 10.3389/fvets.2022.1012462. PMC9731804 36504838

